# A Case of Extensive Thromboembolism as a First Presentation of a Pheochromocytoma: Pathophysiologic Insights and Strategic Surgical Intervention

**DOI:** 10.7759/cureus.96236

**Published:** 2025-11-06

**Authors:** Dinesh Nirmal, Shalini Aurora, Hussam Alkaissi, Ugonna Nanna, Maria Fonseca-Mora, Lina Soni, Samy I. McFarlane

**Affiliations:** 1 Internal Medicine, SUNY (State University of New York) Downstate Health Science University, Brooklyn, USA; 2 Surgery, Kings County Hospital, Brooklyn, USA; 3 Endocrinology, Diabetes and Metabolism, National Institutes of Health (NIH), Bethesda, USA; 4 Endocrinology, SUNY (State University of New York) Downstate Health Science University, Brooklyn, USA; 5 Internal Medicine/Endocrinology, SUNY (State University of New York) Downstate Health Science University, Brooklyn, USA

**Keywords:** acute coronary syndrome, inflammation, pheochromocytoma, protein s deficiency, pulmonary embolism

## Abstract

Pheochromocytomas are rare and often underdiagnosed neuroendocrine tumors characterized by excessive catecholamine production, typically presenting in the fourth to fifth decades of life. While the classic presentation includes labile hypertension and a triad of headaches, palpitations, and sweating, thromboembolism as an initial manifestation of pheochromocytoma is exceedingly rare. In this case report, a 78-year-old male patient with cardiovascular risk factors initially presented with a clinical picture consistent with acute coronary syndrome. However, further clinical workup revealed extensive bilateral pulmonary emboli and an incidental adrenal mass. Subsequent biochemical testing and metaiodobenzylguanidine (MIBG) scintigraphy confirmed the diagnosis of pheochromocytoma. A concurrent thromboembolic workup revealed an underlying protein S deficiency. The patient was managed medically with alpha- and beta-adrenergic blockade. Following hemodynamic stabilization, the patient underwent successful laparoscopic adrenalectomy. Postoperative follow-up demonstrated complete resolution of symptoms and near normalization of biochemical markers. In this report, the pathogenesis of thromboembolism in pheochromocytoma is discussed, highlighting both the clinical features and the sophisticated surgical approach to this complex disorder.

## Introduction

Pheochromocytomas, originating from the chromaffin cells of the adrenal medulla, are notable for their ability to secrete catecholamines in an episodic or sustained manner, resulting in a wide range of clinical manifestations. Traditionally recognized by the classic triad of headache, sweating, and palpitations, alongside hypertension, these tumors pose a significant diagnostic challenge due to their rare occurrence and varied presentation [1].

In recent years, with the widespread use of cross-sectional imaging, up to 5-7% of adrenal incidentalomas have been found to be pheochromocytomas [1-3]. While many are hormonally silent, missed diagnoses carry the risk of peri-procedural hypertensive crisis, underscoring the importance of biochemical screening in all adrenal incidentalomas before surgical or invasive procedures. Our case highlights a pheochromocytoma discovered incidentally during work-up for extensive thromboembolism, adding to the body of evidence that adrenal incidentalomas can present in clinically unexpected ways.

In this report, a unique presentation of pheochromocytoma was diagnosed, manifesting with symptoms mimicking an acute cardiovascular event and complicated by an extensive prothrombotic state, leading to bilateral pulmonary embolism. This case highlights the complexities and diagnostic challenges posed by pheochromocytoma, emphasizing its unusual presentation as a "natural stress test due to catecholamine release" and its association with thromboembolic events [2-7]. This report seeks to contribute to the broader understanding of pheochromocytoma's clinical spectrum and to underscore the importance of considering this diagnosis in patients presenting with unexplained symptoms and signs of catecholamine excess, particularly in the presence of a prothrombotic event [2].

## Case presentation

A 78-year-old man with hypertension, type 2 diabetes mellitus, chronic kidney disease stage 3, and no family history of coagulopathy presented with a one-day history of worsening shortness of breath and central chest pain exacerbated by exertion and alleviated by rest. The patient's vital signs were notable for a heart rate of 94 beats per minute (bpm) with a regular rhythm and an elevated blood pressure of 165/81 mmHg. Electrocardiogram (EKG) and echocardiography showed no acute changes or abnormalities. Initial laboratory tests revealed a mildly elevated lactate level of 1.7 mmol/L, with lateralized serial troponin levels.

**Figure 1:**
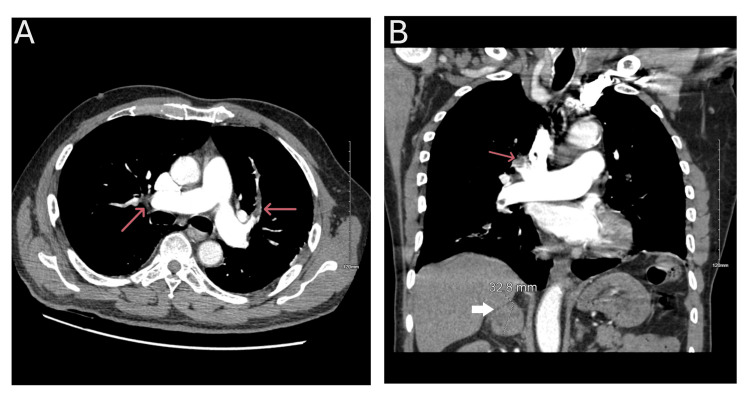
Computed tomography (CT) angiogram of the chest showing extensive bilateral pulmonary emboli with central pulmonary artery enlargement. A. Transverse section showing embolus in the left and right pulmonary arteries (red arrows). B. Coronal section showing an embolus at the pulmonary artery bifurcation (red arrow) and the right adrenal pheochromocytoma (white arrow).

**Figure 2:**
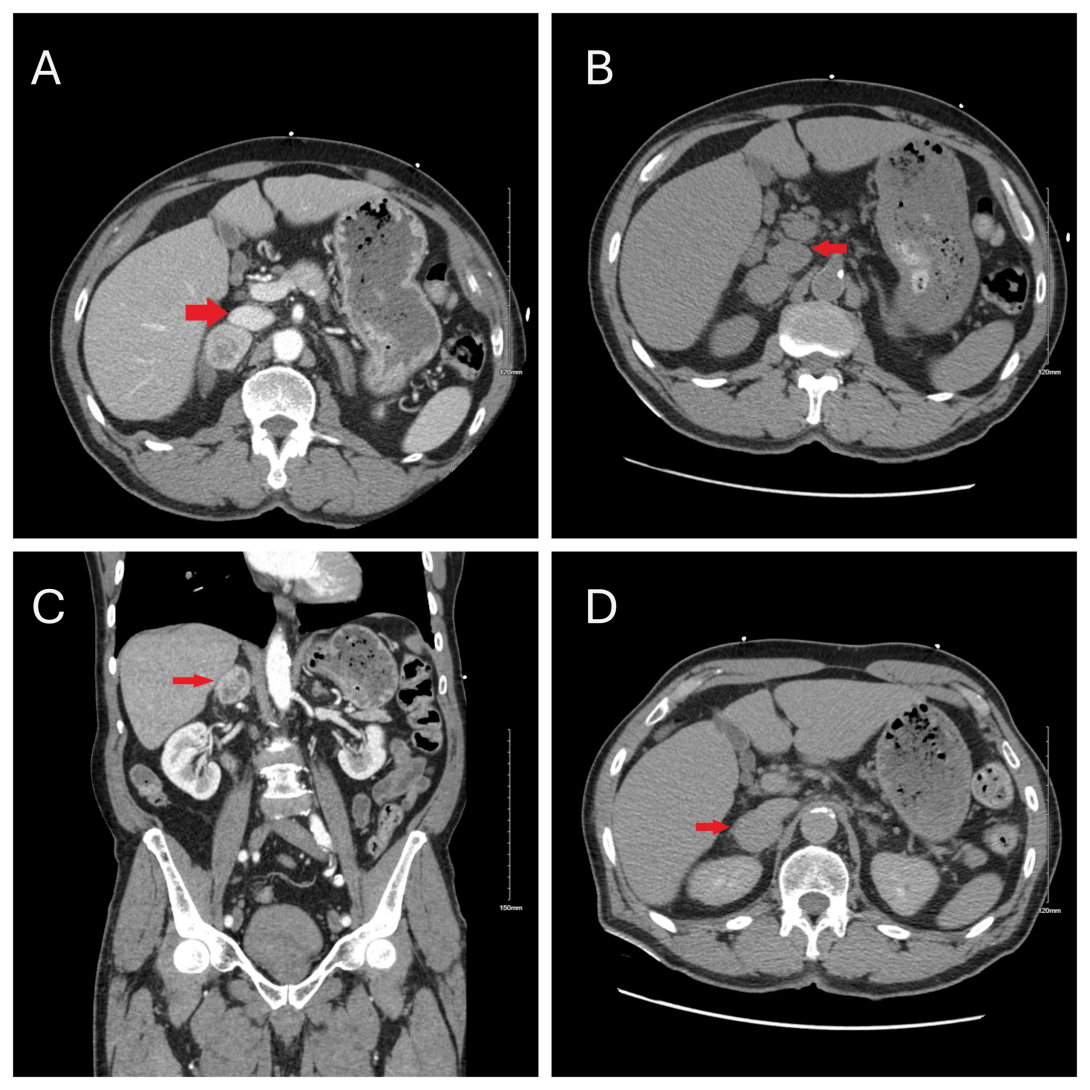
: Computed tomography (CT) scan of the abdomen demonstrating a right adrenal mass (red arrow).

A chest computed tomography (CT) angiogram identified extensive bilateral pulmonary emboli, predominantly on the right side, and enlargement of the central pulmonary artery without right ventricular strain (Figure 1). Additionally, an incidental adrenal mass was noted. Further characterization of the adrenal mass via CT abdomen revealed a right heterogeneous adrenal mass measuring 3.5x3.1x2.6 cm with specific imaging features suggestive of pheochromocytoma, including pre-contrast Hounsfield units (HU) of 40, hyperenhancement to HU 120 on arterial phase with rapid washout to HU 66, leading to a provisional diagnosis (Figure 2).

**Figure 3:**
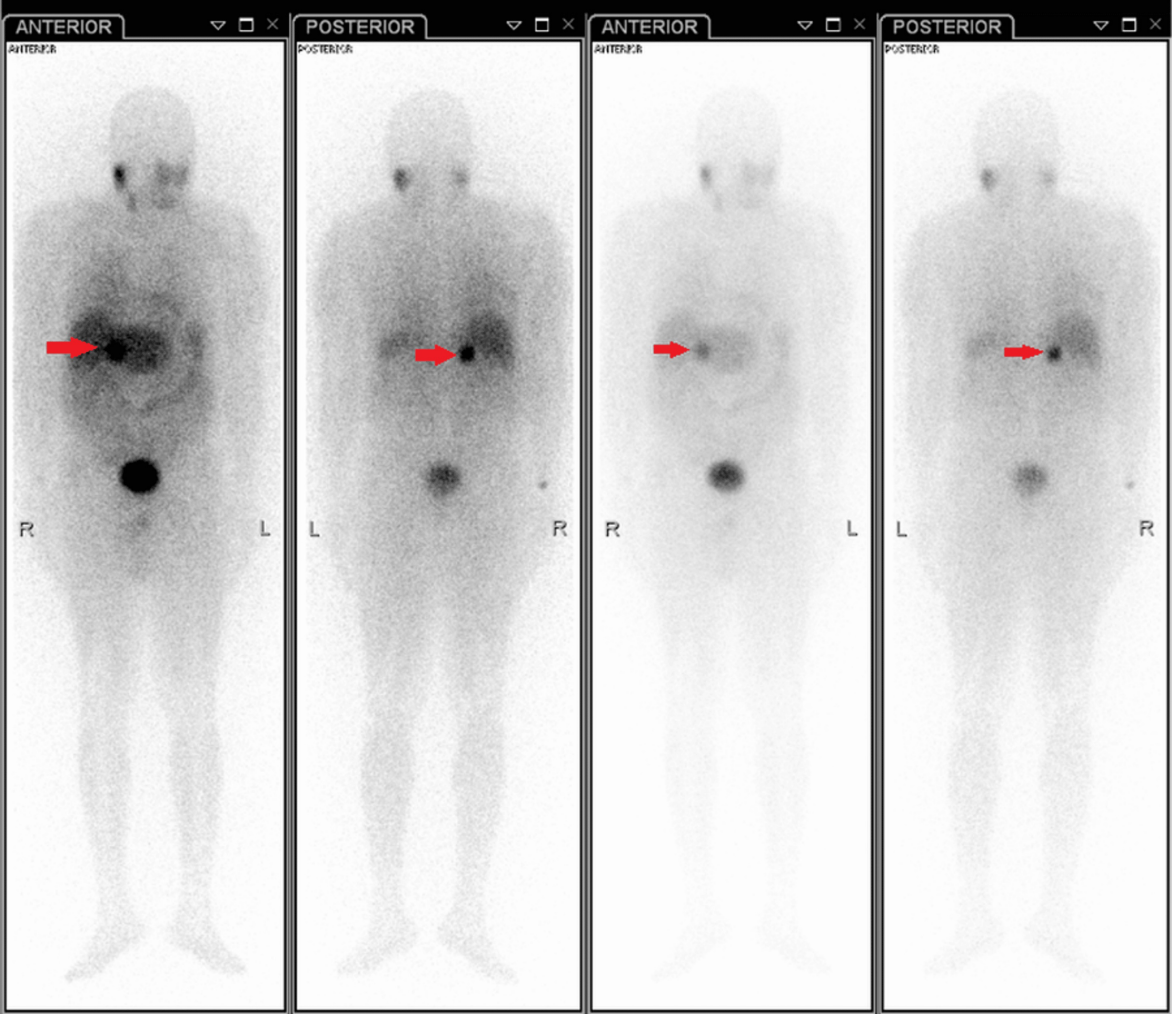
Metaiodobenzylguanide (MIBG) scan showing increased radiotracer uptake in the right suprarenal gland. Red arrows indicate the right adrenal gland demonstrating increased MIBG uptake.

A metaiodobenzylguanidine (MIBG) scan on Day 11 after admission demonstrated increased uptake in the right adrenal gland, suggestive of pheochromocytoma, with the remaining increased activity representing normal physiological uptake (Figure 3). Markedly elevated 24-hour urine metanephrine levels provided the biochemical confirmation of pheochromocytoma at 859 ug/24 hours (normal range: 58-276 ug/24 hours), and normetanephrine levels at 3326 ug/24 hours. (normal range: 156-729 ug/24 hours), with the total volume, reported as 2400 mL for both tests. Additionally, the urine metanephrine-creatinine ratio was elevated to 2.9 (normal value <1). These findings, coupled with the CT and MIBG scan features, confirmed the biochemical diagnosis of pheochromocytoma. The patient remained normotensive and asymptomatic throughout hospitalization. Management began with phenoxybenzamine, followed by the addition of propranolol for alpha and beta-blockade, respectively, to prepare for surgical excision of the tumor.

**Figure 4:**
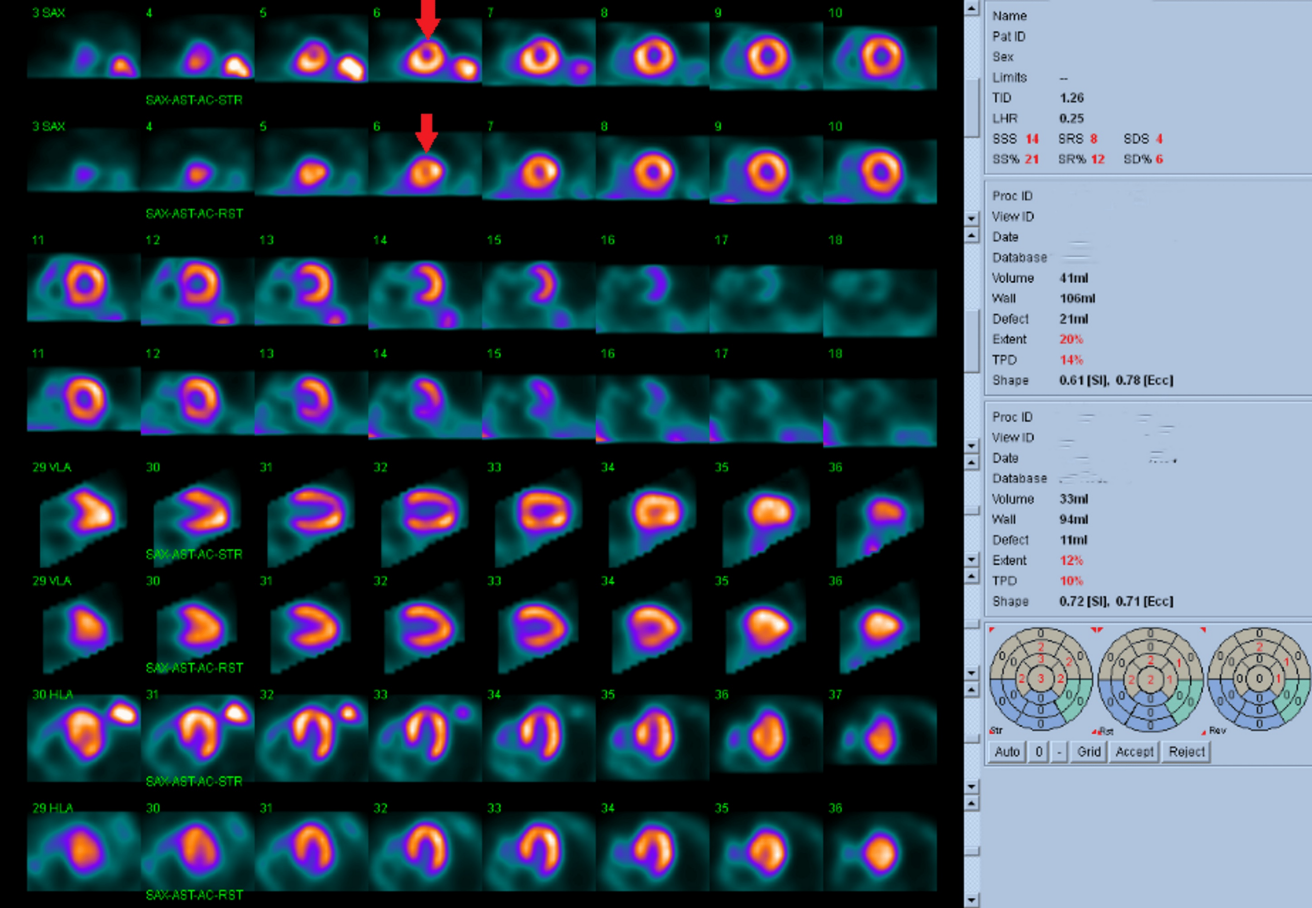
Myocardial perfusion SPECT demonstrating normal perfusion and hyperdynamic left ventricular function. Images demonstrate homogeneous tracer uptake without fixed or reversible perfusion defects, consistent with normal perfusion. Red arrowsindicate the markedly small left ventricular cavity at stress and rest, consistent with hyperdynamic function. Quantitative analysis confirmed a left ventricular ejection fraction >90%. SPECT: single-photon emission computerized tomography

A nuclear cardiac perfusion scan performed on Day 17 showed no EKG evidence of ischemia, normal myocardial perfusion imaging without ischemic perfusion defects or areas of infarction, a hypotensive blood pressure response to stress, and a hyperdynamic left ventricular ejection fraction of 76%, which was an expected response to the medication-induced alpha blockade (Figure 4).

After the initial hospital discharge and before undergoing adrenalectomy, the patient underwent additional laboratory testing to assess underlying hypercoagulability. Notably, the protein S activity was found to be decreased by 43% (reference: 57.6-123.3%), and free protein S antigen was also reduced to 53% (reference: 67-141%). Other coagulation studies, including protein C activity (106%, reference: 70-140%) and anti-thrombin III (87%, reference: 83-128%), were within normal limits. The patient’s erythrocyte sedimentation rate (ESR) was elevated at 42 mm/hour, suggesting an underlying inflammatory state. Prothrombin time (PT), activated partial thromboplastin time (aPTT), and INR were normal. These findings raised concerns for an acquired protein S deficiency, potentially due to an inflammatory acute-phase response related to the pheochromocytoma.

**Figure 5:**
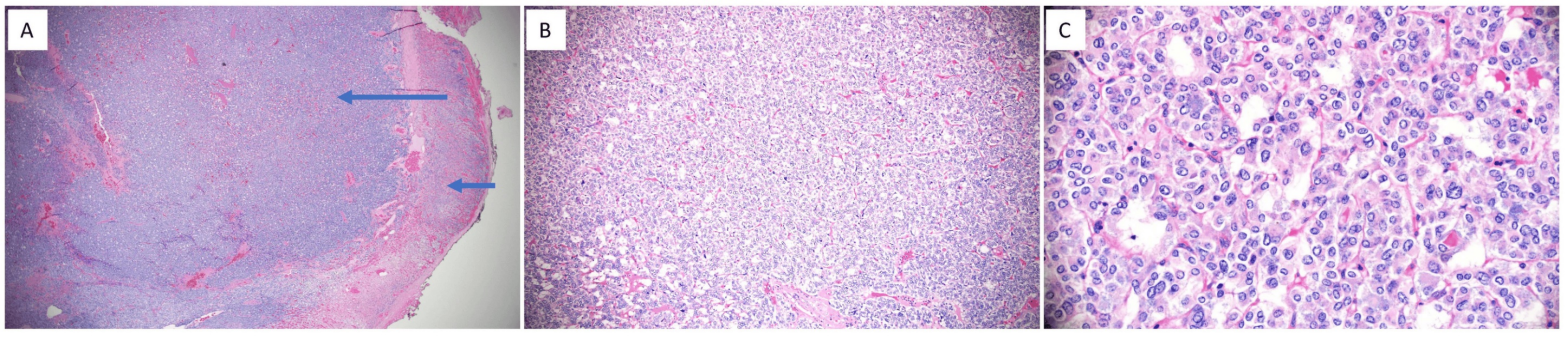
Histologic and cytopathologic analysis of the excised tumor. The tumor (long arrow) is cellular and partially encapsulated, with adjacent compressed adrenal gland tissue (short arrow) (A, x20).  The tumor cells are arranged in a Zellballen pattern, featuring small nests surrounded by fine vasculature (B, x100).  Cytologically, the polygonal tumor cells show abundant amphophilic granular cytoplasm, pale or vesicular chromatin, and indistinct nucleoli (C, x400).

**Figure 6:**
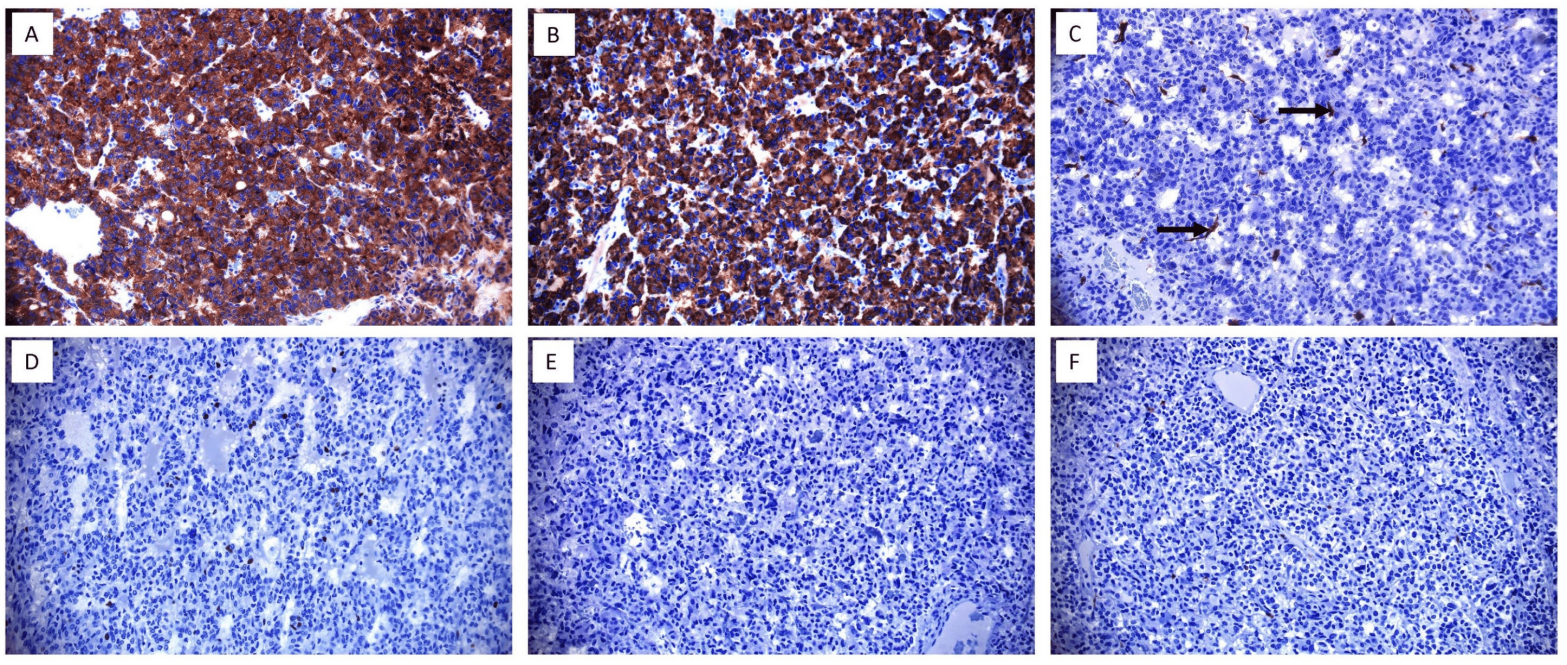
Immunohistochemical staining of pheochromocytoma. (A) Tumor cells showing diffuse positivity for synaptophysin; (B) Tumor cells positive for chromogranin; (C) S100 highlighting sustentacular cells (arrows); (D) Ki-67 proliferative index up to 4%; (E) Tumor cells negative for Melan-A; (F) Tumor cells negative for calretinin.

After attaining appropriate alpha and beta blockade, the patient successfully underwent laparoscopic adrenalectomy. The excised tumor was sent for histologic, cytopathologic, and immunohistochemical analysis (Figures 5, 6). The procedure was uncomplicated; however, the patient required a nitroglycerin (NTG) infusion and ICU admission for blood pressure optimization, with the NTG infusion being weaned off over 10 days. A week following the procedure, serum and urine metanephrine levels were re-measured, demonstrating a significant decrease (Table 1).

**Table 1:** Plasma and urinary catecholamine metabolite levels before and seven days after adrenalectomy. Preoperative values were markedly elevated, while postoperative levels normalized, consistent with biochemical resolution following resection of pheochromocytoma.

	Plasma		Urine			
	Normetanephrine (pg/ml)	Metanephrine (pg/ml)	Normetanephrine (mcg/L)	Normetanephrine (24 hours) (mcg/24 hours)	Metanephrine (mcg/L)	Metanephrine (24 hours) (mcg/24 hours)
Preoperative (pg/ml)	1327.8	336.2	1386	3326	358	859
7 days postoperative	142.0	28.4	652	1255	70	135
Reference ranges	0-285.2	0-88.0	N/A	156-729	N/A	58-276

At the time of writing this report, the patient was being followed as an outpatient for over six months while living symptom-free.

## Discussion

Pheochromocytomas are neuroendocrine tumors that pose unique challenges in diagnosis and management, primarily due to their variable clinical presentation and the potent effects of catecholamine secretion [1-4]. This case highlights the complex interplay between the pathophysiological aspects of the tumor and its clinical implications, including the paradoxical presentations and prothrombotic complications observed.

Pathophysiology and clinical implications

The pathophysiology of pheochromocytoma centers around the tumor's ability to produce excessive catecholamines, which are powerful vasoactive substances. These catecholamines, including epinephrine and norepinephrine, can induce a wide range of cardiovascular, metabolic, and neuropsychiatric effects. The excessive secretion of these substances can lead to episodic hypertension, palpitations, and a state of hypermetabolism, as well as less common presentations like exertional chest pain and dyspnea, as observed in our patient. The case demonstrates the critical role of catecholamines in mimicking pharmacological stress tests, highlighting the tumor's capability to induce cardiovascular manifestations that can mimic other common conditions.

Furthermore, the prothrombotic state associated with pheochromocytoma is an essential aspect of its pathophysiology. Multiple mechanisms contribute to this hypercoagulable state.

Catecholamine Excess

Chronic high levels of norepinephrine/epinephrine can affect coagulation and the vasculature. Catecholamines may promote platelet activation and cause intense vasoconstriction and hypertension, leading to endothelial injury. Severe hypertension and shear stress can damage the endothelium, exposing prothrombotic surfaces (Virchow’s triad). Adrenergic surges can also raise levels of von Willebrand factor and factor VIII (as seen in stress responses), tipping the hemostatic balance towards clotting [5,7].

*Inflammatory Cytokines (*interleukin (*IL)-6 and Acute-Phase Response)*

Pheochromocytomas occasionally lead to the production of cytokines, such as IL-6, or provoke an inflammatory acute-phase reaction in the host. IL-6-producing pheochromocytomas are well-documented and tend to present with fever, elevated inflammatory markers, anemia, and thrombocytosis, rather than the typical tachycardia/hypertension [8,9]. IL-6 is a potent driver of hepatic acute-phase protein synthesis; it raises fibrinogen, C-reactive protein (CRP), and complement factors, while lowering albumin. Notably, fibrinogen is both an inflammatory marker and a coagulation factor; elevated fibrinogen promotes fibrin formation and blood hyperviscosity [9]. In IL-6-secreting pheochromocytomas, extreme CRP and fibrinogen levels have been observed alongside very high ESRs (ESR > 100 mm/hour) [8,9]. This cytokine storm environment favors thrombosis. IL-6 can also induce megakaryocytosis and thrombocytosis, increased platelet production, which is seen in such patients (platelet counts are typically >500-600k) [8,9]. An excess of platelets further contributes to a prothrombotic state.

Effects on Protein S (Acquired Protein S Deficiency)

One consequence of the acute-phase reaction is a reduction in protein S, an anticoagulant protein. IL-6 stimulates production of C4b-binding protein (C4b-BP), a circulating protein that binds to protein S. When C4b-BP levels rise, they sequester more protein S, effectively lowering the free (active) protein S available to act as a cofactor for protein C. This phenomenon is known as an acquired protein S deficiency. By analogy, a pheochromocytoma that triggers high IL-6, or an acute-phase response, could lead to a similar decrease in protein S activity. Thus, pheochromocytoma may indirectly decrease protein S levels through cytokine-mediated acute-phase changes rather than a direct effect on protein S synthesis. In most reported pheochromocytoma cases with thrombosis, intrinsic thrombophilia (inherited protein S deficiency, factor V Leiden, etc.) was not identified, suggesting the low protein S activity is usually acquired and reversible [10,11].

Rouleaux Formation and Elevated ESR

An elevated ESR in pheochromocytoma patients can be partly attributed to rouleaux formation resulting from hypercoagulability. ESR rises when red blood cells form stacks (rouleaux) and settle quickly, which occurs in the presence of high levels of fibrinogen or immunoglobulins. Fibrinogen, being an acute-phase protein, is frequently elevated in active pheochromocytoma (especially if there is an IL-6-mediated response) [9]. This fibrinogen can bind to erythrocytes and promote their aggregation, accelerating the ESR as a reflection of hypercoagulability. In clinical settings, a very high ESR in a pheochromocytoma patient (in the absence of infection or malignancy elsewhere) often correlates with the extent of inflammation and coagulopathy induced by the tumor [9].

The prothrombotic state observed in this case, including the decreased protein S activity and elevated ESR, is consistent with the inflammatory and catecholamine-driven changes associated with pheochromocytoma. These findings emphasize the importance of recognizing pheochromocytoma as a potential driver of hypercoagulability, particularly in patients presenting with unexplained thromboembolic events.

Management strategies: a comprehensive approach

The management of pheochromocytoma requires a multifaceted approach, incorporating both medical and surgical strategies to address the tumor's effects and mitigate complications. Initial management focuses on controlling hypertension and symptoms of catecholamine excess with alpha-blockade, as demonstrated with phenoxybenzamine in this case, followed by beta-blockade to manage tachycardia and arrhythmias. This pharmacologic approach is critical for stabilizing the patient before definitive surgical intervention, which remains the cornerstone of treatment for pheochromocytoma [1,4].

Surgical excision of the tumor offers the potential for a cure, but it requires careful preoperative preparation and intraoperative management to avoid catecholamine-induced hemodynamic instability. The role of minimally invasive surgical techniques, such as laparoscopic adrenalectomy, has grown, offering advantages in terms of reduced postoperative pain, shorter hospital stays, and quicker recovery. However, the choice of surgical approach must be individualized, considering the tumor's size, location, and the presence of metastatic disease [1,4,12].

In addition to addressing the tumor directly, management strategies must also encompass the treatment of complications, including the prothrombotic state. Anticoagulation therapy may be warranted in patients with significant thromboembolic events, as seen in our case. The decision to initiate anticoagulation must be balanced against the risk of bleeding, particularly in patients undergoing surgical intervention [1,4].

Implications for clinical practice

This case highlights the importance of a multidisciplinary approach in managing pheochromocytoma, which involves collaboration among endocrinologists, cardiologists, anesthesiologists, and surgeons. It also emphasizes the need for clinicians to maintain a high index of suspicion for this tumor in patients presenting with unexplained cardiovascular symptoms or thromboembolic events. Early recognition and comprehensive management are crucial in reducing the morbidity and mortality associated with this condition.

## Conclusions

In this report, a rare case of pheochromocytoma presented as bilateral extensive thromboembolism. This highlights the significant challenge in clinical medicine that requires an in-depth understanding of its pathophysiology and a comprehensive management strategy to address its multifaceted presentations and complications. This case also underscores that when thromboembolic events coexist with an adrenal incidentaloma, clinicians should consider pheochromocytoma as a unifying diagnosis rather than attributing the findings to coincidence. This case contributes to the literature by illustrating the complexities involved in diagnosing and managing pheochromocytoma, highlighting the importance of vigilance and a multidisciplinary approach to care. Further research into optimizing management protocols and exploring new therapeutic targets will continue to improve outcomes for patients with this rare but impactful condition.
